# Ultrasound stimulation for non-invasive visual prostheses

**DOI:** 10.3389/fncel.2022.971148

**Published:** 2022-08-03

**Authors:** Jaya Dilip Badadhe, Hyeonhee Roh, Byung Chul Lee, Jae Hun Kim, Maesoon Im

**Affiliations:** ^1^Brain Science Institute, Korea Institute of Science and Technology (KIST), Seoul, South Korea; ^2^Division of Bio-Medical Science & Technology, KIST School, University of Science and Technology (UST), Seoul, South Korea; ^3^School of Electrical Engineering, College of Engineering, Korea University, Seoul, South Korea; ^4^KHU-KIST Department of Converging Science and Technology, Kyung Hee University, Seoul, South Korea; ^5^Sensor System Research Center, Korea Institute of Science and Technology (KIST), Seoul, South Korea

**Keywords:** ultrasound stimulation, neuromodulation, artificial vision, vision restoration, visual prosthesis

## Abstract

Globally, it is estimated there are more than 2.2 billion visually impaired people. Visual diseases such as retinitis pigmentosa, age-related macular degeneration, glaucoma, and optic neuritis can cause irreversible profound vision loss. Many groups have investigated different approaches such as microelectronic prostheses, optogenetics, stem cell therapy, and gene therapy to restore vision. However, these methods have some limitations such as invasive implantation surgery and unknown long-term risk of genetic manipulation. In addition to the safety of ultrasound as a medical imaging modality, ultrasound stimulation can be a viable non-invasive alternative approach for the sight restoration because of its ability to non-invasively control neuronal activities. Indeed, recent studies have demonstrated ultrasound stimulation can successfully modulate retinal/brain neuronal activities without causing any damage to the nerve cells. Superior penetration depth and high spatial resolution of focused ultrasound can open a new avenue in neuromodulation researches. This review summarizes the latest research results about neural responses to ultrasound stimulation. Also, this work provides an overview of technical viewpoints in the future design of a miniaturized ultrasound transducer for a non-invasive acoustic visual prosthesis for non-surgical and painless restoration of vision.

## Introduction

Permanent blindness can be caused by diverse visual diseases such as retinitis pigmentosa (Yue et al., 2016), age-related macular degeneration (Jackson et al., 2002), and optic neuritis (Toosy et al., 2014). Although these diseases are incurable (Lo et al., 2020), the implantation of visual prostheses is one of the available options for vision restoration (Shepherd et al., 2013; Fernandez, 2018; Im and Kim, 2020; Kim et al., 2022). Recently, electrical stimulation is used for the activation of neurons in the retina, optic nerve, lateral geniculate nucleus, and visual cortex. For example, Argus II (Humayun et al., 2009; Farvardin et al., 2018), Alpha IMA/AMS (Zrenner et al., 2011; Stingl et al., 2015), and Orion (Beauchamp et al., 2020) have been successfully implanted onto/underneath the human retina or at the visual cortex, respectively; and they have shown clinically promising results. However, there are still several challenges to be overcome for more useful visual prostheses. Those issues include slow perception of artificial vision, limited numbers of pixels (i.e., electrodes), invasiveness of microelectrodes, and so on.

Among those issues, the invasive implantation requires considerable surgical costs and may show surgical side effects. Many efforts have recently been made to implement non-invasive visual prostheses, including the investigation of optogenetics (Barrett et al., 2014), photoswitches (Tochitsky et al., 2016), and artificial opsins (Park et al., 2018; Berry et al., 2019). In the past decade, ultrasound-based neuromodulation in the brain and peripheral nervous system has gathered huge attention due to its non-invasiveness, deep penetration power through the skull with minimal neural tissue damage (Lee et al., 2016), and sub-mm focusing capability (Kim et al., 2021). Ultrasound has long been used for both diagnostic imaging (Wells, 2006) and therapeutic treatment (Steiss and McCauley, 2004) including cancer tissue destruction (Hsiao et al., 2016), proving its safety. In recent years, ultrasound stimulation technology (UST) has been used for neuromodulation of the nerve cells of the retina (Yue et al., 2016; Jiang et al., 2019; Lo et al., 2020) and of the visual cortex (Lee et al., 2016; Lu et al., 2021). The purpose of the present study is to overview recent studies regarding UST aiming for vision restoration as well as to discuss future perspectives on the development of acoustic visual prostheses.

## Working principle of ultrasound stimulation

**FIGURE 1:**
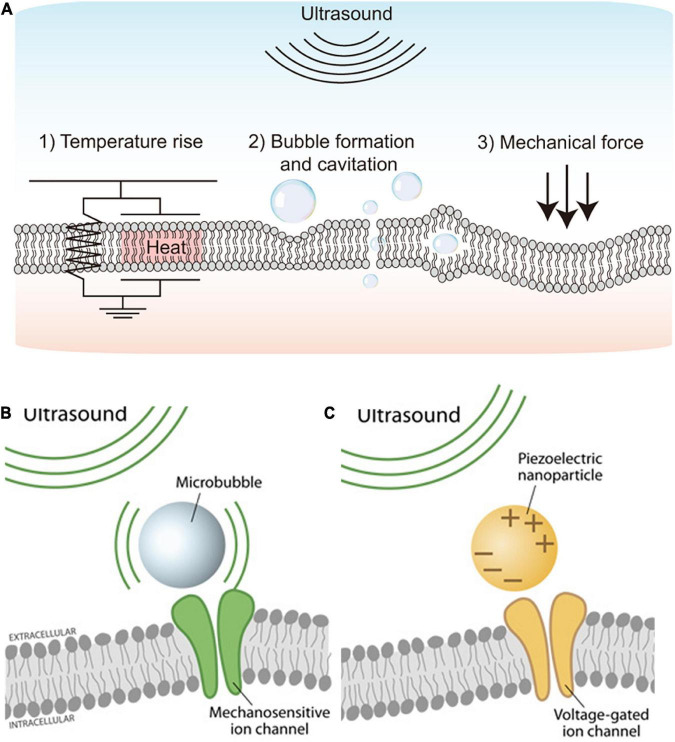
Schematics showing the ultrasound stimulation mechanism and ultrasound stimulation methods. **(A)** Physical mechanisms of ultrasound-based excitation on neurons. Biophysical effects of ultrasound such as (1) Temperature rise, (2) Bubble formation and cavitation, and (3) Mechanical force. Adapted from Yoo et al. (2022). **(B)** Microbubble-assisted ultrasound stimulation of mechanosensitive channels. Adapted from Rivnay et al. (2017). **(C)** Piezoelectric nanoparticle-assisted ultrasound stimulation of voltage-sensitive ion channel. Adapted from Rivnay et al. (2017).

The application of ultrasound can make the following three physical events: (1) Increase in temperature, (2) Bubble formation and cavitation, and (3) Acoustic radiation force (Yoo et al., 2022). It has been known that neural activities can be induced by the aforementioned physical changes (O’Brien, 2007; Tyler et al., 2008; Plaksin et al., 2016). First, it had been demonstrated that high-intensity (>1 W/cm^2^) ultrasound can evoke action potentials in peripheral neuronal cells due to the increased excitability at higher temperature (Figure 1A1; Tsui et al., 2005; Yoo et al., 2022). Because too much heat damages tissue by protein denaturation and decreases synaptic transmission (Dalecki, 2004; O’Brien, 2007), low-intensity (<500 mW/cm^2^) ultrasound had been tested for neuromodulation without hyperthermia effect (Dalecki, 2004; O’Brien, 2007). Interestingly, the neural activities were also modulated even with a minimal temperature rise, making the thermal mechanism less convincing.

Second, cavitation is a non-thermal effect caused by ultrasound (Figure 1A2), which directly modulates ion channels as well as the plasma membrane for neuromodulation (Fomenko et al., 2018). Cavitation makes gas bubbles forming, oscillating, and possibly collapsing within the tissue, resulting in stimulation of action potentials and synaptic transmission by deforming the bilayer lipid membrane (Krasovitski et al., 2011). The cavitation can happen by ultrasound waves at low frequency (1–3 MHz); however, acoustic pulses at higher frequencies (>4 MHz) are difficult to be used because oscillations in bubbles are difficult to be maintained during cavitation (Menz et al., 2019).

Lastly, acoustic radiation force is the most widely accepted potential physical mechanism of ultrasound-based neuromodulation (Figure 1A3). The mechanical force generated by steady acoustic pressure on the target neuron stretches the cell membrane and results in conformation and deformation of mechanosensitive ion channels in the cell membrane (Fomenko et al., 2018; Menz et al., 2019; Qian et al., 2022).

Mechanosensitive ion channels are transmembrane proteins that can detect and respond to mechanical stimuli; they can act as mechanosensitive nanovalve and provide a neuronal response to microbubbles generated by applied ultrasound (Figure 1B). Stretch-sensitive channels, displacement-sensitive channels, and shear stress-sensitive ion channels are commonly recognized as mechanosensitive ion channels (Morris, 1990). When the ultrasound is applied, mechanosensitive proteins undergo a conformational change that stimulates ion channels (Johns, 2002; Sukharev and Corey, 2004). Several earlier studies have focused on the significance of various mechanosensitive ion channels in various types of neurons (Menz et al., 2017; Ye et al., 2018; Wang et al., 2020; Yoo et al., 2022). Ultrasound has recently been used to trigger mechanosensitive K^+^, Ca^2+^, and Na^+^ channels that are found in the retina and brain (Maingret et al., 1999; Kubanek et al., 2016; Sorum et al., 2021). Those mechanosensitive proteins include MEC-4 (Kubanek et al., 2018), TRPP1/2 (Duque et al., 2022), TRPV1 (Yang et al., 2020), Piezo 1 (Qiu et al., 2019a), MscL (Ye et al., 2018), TRAAK (Sorum et al., 2021), and the K2p family (Zhao et al., 2017). Although it has not been fully understood, those mechanosensitive components are believed to play a critical role in neuromodulation using UST (Ye et al., 2018). To improve cell-type specificity, sonogenetics is also recently introduced (Ibsen et al., 2015; Qiu et al., 2019b; Yoo et al., 2022). The sonogenetics approach is a combination of ultrasound-based neuromodulation with mechanosensitive channel proteins, which can add cell type-specificity to conventional UST.

In addition to the direct activation of mechanosensitive ion channels, voltage-gated ion channels can be indirectly activated by ultrasound stimulation. For example, piezoelectric nanoparticles stimulated by external ultrasound can generate electrical charges in the target tissues, activating voltage-gated ion channels for neuromodulation (Figure 1C). A previous study demonstrated piezoelectric stimulation induces Ca^2+^ influx that helps in neuronal stimulation (Marino et al., 2015). This indirect electrical stimulation assisted by piezoelectric nanoparticles can serve as nano-transducers at both tissue and cell levels (Marino et al., 2017; Rivnay et al., 2017; Cafarelli et al., 2021). Earlier studies have used boron nitride nanotubes (BNNTs), barium titanate nanoparticles (BTNPs), zinc oxide (ZnO) nanowires, and polyvinylidene fluoride-trifluoroethylene (PVDF- TrFE) as piezoelectric nanoparticles (Cafarelli et al., 2021). However, *in vivo* proof of the feasibility and efficacy of piezoelectric nanoparticles-based ultrasound therapy for vision restoration has yet to be demonstrated. Also, more in-depth studies may be required to explore any potential toxicity and long-term biocompatibilities of piezoelectric nanoparticles (Cafarelli et al., 2021). For instance, piezoelectric nanoparticles such as lead zirconate titanate (PZT) are less biocompatible due to the lead element but further, PZT was made more biocompatible by treating its surface with titanium (Sakai et al., 2006).

## Ultrasound stimulation of retina and visual cortex

**FIGURE 2:**
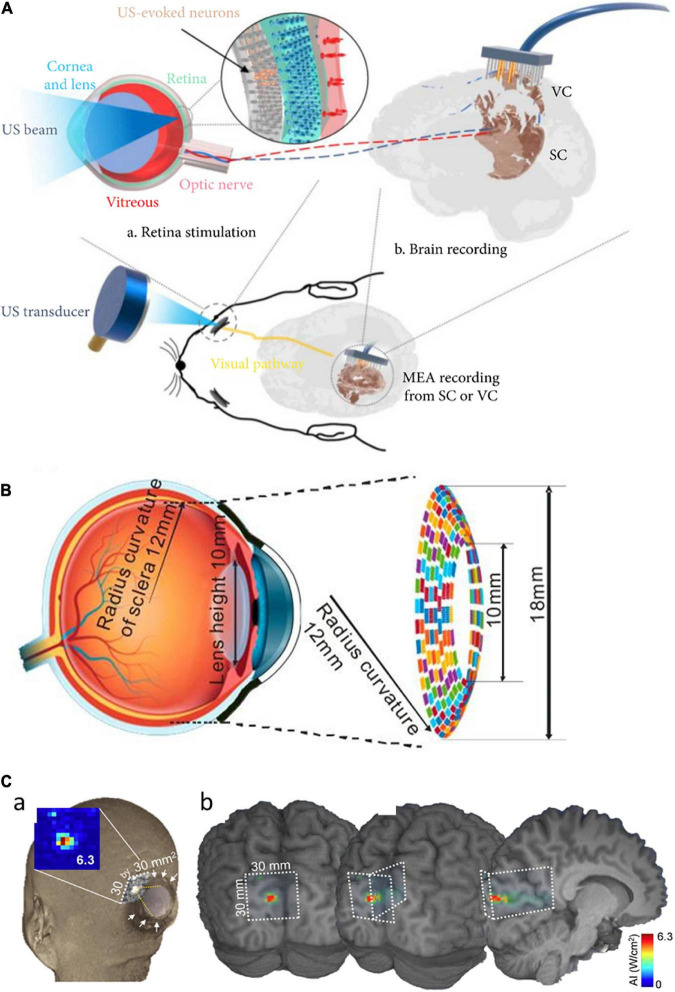
Focused ultrasound treatment for visual restoration. **(A)** Retinal neurons were excited by ultrasound waves which lead to the generation of the neural signal. These signals are transmitted to the brain via the optic nerve and the brain activity is recorded from the visual cortex or the superior colliculus. Adapted from Qian et al. (2022). **(B)** The circular racing array device for ultrasonic stimulation. Adapted from Yu et al. (2019). **(C)** In simulated acoustic intensity profiles, the acoustic focus was effectively projected to the targeted stimulatory site localized in the calcarine fissure (a), and acoustic energy was delivered to the visual cortex (b). Adapted from Lee et al. (2016).

Nowadays, low-intensity focused ultrasound (LIFUS) becomes widely used as a non-thermal, non-invasive approach for generating neuromodulation toward vision restoration (Baek et al., 2017; Fomenko et al., 2018; Jiang et al., 2018, 2019; Yuan et al., 2019). It has been repeatedly demonstrated that FUS can effectively stimulate the neurons *in vitro* (Menz et al., 2017; Cafarelli et al., 2021), *ex vivo* (Blackmore et al., 2019; Menz et al., 2019; Sarica et al., 2022), and *in vivo* (Naor et al., 2012; Lee et al., 2015). For the vision restoration purpose, an acoustic retinal prosthesis (ARP) had been proposed for the first time by demonstrating a FUS neurostimulation of the retinal cells in anesthetized wild-type rats (Naor et al., 2012). The ARP consists of an ultrasound phased array and an external camera with an image processor, connected with the cornea via an acoustic coupling component, to transmit acoustic images onto the retina. Later, a high frequency (43 MHz) ultrasound was used for stimulation of the salamander retina which generates high spatiotemporal resolution and stable visual responses (Menz et al., 2013). The acoustic pulses focused on target cells produced spiking activities like both ON and OFF types of retinal ganglion cells (RGCs) with temporal precision similar to the visual responses. Their spatial resolution was ∼100 μm but higher frequency ultrasound is expected to achieve smaller activation; however, it may damage lens tissues by generating heat. In another study, the responsiveness of RGCs in the rodent retinas to low frequency (2.25 MHz) FUS was systematically investigated (Jiang et al., 2018). Recently, RGC activities were modulated with even lower US frequency (1 MHz) at low-intensity (0.5 W/cm^2^) (Zhuo et al., 2022). The responses of RGCs greatly varied within each cell type as a function of different ultrasound intensities, suggesting neurophysiological properties of RGCs plays an important role in ultrasound responses. Also, the responses to ultrasound stimulation were not the same as those to light stimulation, implying some limitations for high-quality artificial vision. In the work of Jiang et al. (2018), double burst responses to ultrasound stimulation were observed; the latencies of second bursts were comparable to those of the delayed bursts that have been observed in electrical responses (Im and Fried, 2015; Im et al., 2018; Lee and Im, 2019; Kang et al., 2021). This similar temporal property suggests both ultrasound and electric stimulation may share common RGC activation mechanism(s). More recently, *in vivo* stimulation of blind rats’ retina using a 3.1 MHz spherically focused single-element transducer was reported for the first time (Qian et al., 2022). The ultrasound stimulation of the retina in spatial resolution of 250 μm evoked neural signals in the visual cortex (Figure 2A).

Different areas of retinal tissue can be simultaneously stimulated using a multiple-focus ultrasound transducer array (Li et al., 2018). For easy implementation of the ARP, another study proposed a flexible wearable contact lens transducer array that covers the entire pupil, enabling multi-depth stimulation (Gao et al., 2017). The multi-focused phased array transducers can be worn like a contact lens to the external surface of the eyeball. In the simulation study, it has been estimated that acoustic stimulation at 2.5 MHz can stimulate multiple points in the retina with ∼1.3 mm lateral resolution (Gao et al., 2017). But, there was an issue limiting the application of this approach: the lens tissue in the eyeball absorbs ultrasound energy which further elevates the temperature at the treatment site that can be harmful to the eye. To solve this issue, a racing array transducer similar to a contact lens was proposed for the development of ultrasound retinal stimulation (Yu et al., 2019). The racing array transducer was composed of an array of transducer elements aligned on a concave surface and the center part of the transducer was hollow. In the racing array transducer approach (Figure 2B), the ultrasound absorption in the lens was minimized by directly applying the ultrasound to retina tissue without passing through the lens tissue, thereby avoiding retinal damage due to the potential heating. For a wide coverage of the visual field, a large flexible 2D matrix form of a capacitive micromachined ultrasonic transducer (CMUT) array can be one of the promising candidate systems (Tyler et al., 2018). The CMUT also has advantages of high temporal resolution, reduced size of the focal spot sidelobes, as well as easy monolithic integration with microelectronics.

Ultrasound stimulation technology can be applied to not only the retina but also the visual cortex (Ghezzi, 2015; Kim et al., 2015; Naor et al., 2016; Lu et al., 2021). For instance, ultrasound was successfully applied transcranially to the primary visual cortex of sheep, generating electroencephalographic potentials associated with ultrasound stimulation (Lee et al., 2016). The transcranial focused ultrasound (tFUS) approach is known to be safe and effective for transient neuromodulation (Di Biase et al., 2019). Low-intensity tFUS has elicited blood-oxygen-level-dependent (BOLD) responses in the human primary visual cortex and associated visual areas in the visual cortex, which was correlated with the perception of phosphenes (Lee et al., 2016). The on-site acoustic intensity and spatial resolution of the ultrasonic treatment were estimated using a retrospective numerical simulation of acoustic wave propagation through the skull (Figure 2C). While ultrasound has to penetrate the porous skull in the case of the cortical stimulation, the ultrasound wave for the retinal stimulation passes through a clear homogenous soft medium such as aqueous humor. This technically means that the retinal stimulation has benefits to use the non-invasiveness because the ultrasound energy is less attenuated and the pathway of the ultrasound wave can be well predictable to make higher spatiotemporal focal spots. In contrast, the stimulation of intact visual cortex may have advantages over the stimulation of degenerate retina which is known to have significant remodeling (Jones et al., 2016).

In previous studies, only normal animals and humans with no vision impairment were evaluated (Legon et al., 2018; Di Biase et al., 2019). It was quite recent that tFUS stimulation was tested to evoke neuronal activities of the visual cortices of both normal and blind rats (Lu et al., 2021). Another study compared responses to ultrasound stimulation using both Long Evans (LE) and Royal College of Surgeon (RCS) rats (Qian et al., 2022). Intriguingly, RCS (blind) rats showed much bigger response onset latencies and considerably stronger responses than those of LE (normally sighted) rats, probably due to prolonged visual deprivation.

Although tFUS has been used to modulate neuronal activity deep in the brain (Tufail et al., 2011; Legon et al., 2014, 2018; Di Biase et al., 2019; Lu et al., 2021), these approaches have some limitations such as restricted to low-frequency stimulation, low spatial resolution (>3 mm), and no cell-type selectivity. To overcome these issues, sonogenetics approach was explored (Ibsen et al., 2015). For example, a recent sonogenetics study expressed mechanosensitive ion channels (MscL) in the primary visual cortex of rodents; they achieved high spatial (∼100 μm) and temporal (<50 ms) resolution (Cadoni et al., 2021). It has been known that the activation of 100 μm area in diameter would result in the restored visual acuity of 20/400 (Palanker et al., 2020). The increased sensitivity of neuronal cells to UST was observed by heterogeneously expressed MscL, which increases Ca^2+^ influx (Cadoni et al., 2021). Considering these results, the sonogenetic strategy may open a new door for the application of engineered mechanosensitive channels for vision restoration in blind patients.

## Issues to be addressed for the development of successful acoustic visual prostheses

Retinal UST studies (Naor et al., 2012; Qian et al., 2022) showed a spatial resolution similar to that of the Argus II (electrical stimulation system), the first and only FDA approved retinal prostheses. Despite recent promising results of the ultrasound in vision restoration studies (Menz et al., 2017; Lu et al., 2021; Rousou et al., 2021), several issues need to be addressed further for the clinical success of acoustic visual prostheses. First, the mechanism of neuromodulation of ultrasound stimulation should be more comprehensively understood. In particular, it is unclear whether the neuromodulation effect of ultrasound is universal or limited to specific cell types as well as whether the cellular compartment responding to ultrasound is existing in the degenerate retinas (Lo et al., 2020). If the underlying mechanism is revealed, the ultrasound stimulation conditions (e.g., frequency, therapy duration, duty cycle, and intensity) can be further optimized to efficiently modulate neuronal activities and/or enhance spatiotemporal resolution. For example, it has been known that the physical effect varies with changes in acoustic frequency: cavitation decreases with increasing frequency, and acoustic radiation force increases with increasing frequency (Fan et al., 2021).

Second, additional studies are essential to determine the long-term reliability and/or safety of repetitive application of ultrasonic stimulation. To date, it has been reported that repeated application of ultrasound for 36–48 h did not alter fine membrane structures (Tyler et al., 2008). However, according to the FDA safety guidelines, the acoustic intensity on the eye should be less than 50 mW/cm^2^, which is substantially lower than the safety threshold for other organs (720 mW/cm^2^) (Leary et al., 2008). Therefore, long-term mechanical damages due to continuous repetitive stimulation for dynamic artificial vision need to be additionally investigated. Also, the elevation of temperature can be unsafe for the eye. Typically, ultrasound transducers consume high power; therefore, future ultrasound-based visual prostheses must enable low-power neuromodulation not only for the light-weight batteries but also for less heat generation. Several recent attempts have provided some solutions for the power issue with the wireless energy transfer such as ultrasonic power delivery (Jiang et al., 2022).

Third, although all the parts of the LIFUS system can be mountable on the eye with wireless power transfer, further miniaturization of the whole UST system would be necessary because the size of the external stimulator is highly dependent on the stimulating power. For example, in the case of the high intensity focused ultrasound (HIFUS) system, which needs a high voltage power supply to generate high pressure from an ultrasonic transducer, additional electronics such as power amplifiers may hinder the implementation of the wearable device size. However, technological advances for increased energy efficiency in transducers, wireless power transfer, and batteries are all expected to not only reduce the size of external stimulators but also lengthen operating hours. Indeed, recent publications have demonstrated a CMUT array with monolithically integrated circuits can be chronically implantable even on a rat (Seok et al., 2021a,b). A 2D ultrasonic transducer array system would be preferred to create multi-focused patterns of activities rather than a single transducer with a raster scanning (Lo et al., 2020), which requires lots of extra bulky moving components.

## Conclusion

Thanks to the non-invasiveness, UST has a potential to be a burgeoning field in neural prosthetics. Ultrasound approaches are still in their early stages, and an understanding of interaction with cells/tissues will be important for its successful advancement in the future. Both animal and human studies affirm ultrasound neuromodulation can be helpful for simple and non-invasive interference of impaired visual functions (Lee et al., 2016; Qian et al., 2022). Despite the promising feasibility results, many questions regarding the technological framework of the UST are still unanswered. Most importantly, the working principle behind the biological transduction of ultrasound is yet to be completely understood whether related to particular cell types. There are not many reports about *in vivo* demonstration of vision restoration. However, due to its non-invasiveness and potential for high-resolution stimulation, UST can be widely preferred. It is further expected that the use of engineered neuronal cells with mechanosensitive ion channels would provide high spatiotemporal resolution. Future research work in the abovementioned areas is needed to make the UST to be clinically applicable in blind patients.

## Author contributions

JB and HR contributed to drafting the manuscript. MI conceived the study and revised the manuscript. BL and JK revised the manuscript. All authors read and approved the final manuscript.
